# A spotlight on the MSc Cancer at UCL, 15 years on

**DOI:** 10.3389/fonc.2025.1610068

**Published:** 2025-06-18

**Authors:** Sarah Koushyar, John A. Hartley

**Affiliations:** Department of Cancer Education, UCL Cancer Institute, University College London, London, United Kingdom

**Keywords:** postgraduate education, MSc Cancer, cancer education, curriculum evolvement, educational practices

## Abstract

Cancer remains one of the most significant global health challenges, thus education on the most recent advancements in the understanding of cancer science, diagnosis and treatment is vital. The University College London (UCL) MSc Cancer is now in its 15^th^ year. This program not only equips students with the comprehensive knowledge of cancer biology and therapeutics but provides cutting edge skills needed to succeed beyond the MSc. The curriculum integrates the fundamentals of normal cell and cancer biology through to the cancer patient, creating a bench to bedside educational pipeline. This article gives a holistic overview of the MSc Cancer curriculum, the research-led educational practices being carried out, the research opportunities that branch from this MSc and the career prospects of MSc Cancer graduates. Further detailed information on the MSc Cancer can be found here: Cancer MSc | Prospective Students Graduate - UCL – University College London.

## 1 Introduction

Globally, cancer is the second leading cause of death, with an estimation of 9.6 million deaths in 2020 (WHO, 2024). Although there have been significant advancements in both the understanding of cancer and therapeutic interventions, gaps and challenges remain in our knowledge regarding cancer prevention, initiation, progression, and treatment (1). Thus, the MSc Cancer was devised to provide current knowledge to address these gaps and challenges educating the future generation of cancer scientists, whilst learning in a world class Institute.

The MSc Cancer was first introduced in 2009 to fulfil the need for a postgraduate qualification in the rapidly expanding field of cancer research and treatment. Prior to this at UCL, an MSc in Molecular Medicine offered a popular specialist module in Molecular Oncology. Until 2012, the MSc Cancer ran in parallel with a small highly specialized MSc in Radiation Biology (2).

The MSc Cancer is not an Oncology course, but a course that examines the research which underpins our understanding of cancer as a disease and the approaches to diagnosis and therapy. Over the last 15 years, this course has been pioneering the field of cancer education, with a continuous focus on innovations in translational cancer medicine, which is one of the strengths of the over 400 staff within the UCL Cancer Institute (CI). This article reflects on how the curriculum has evolved, the research opportunities that have stemmed from it, our educational practices to enhance student learning, and the career prospects for our graduates.

At the time of inception, the MSc Cancer was unique with both its title and curriculum, and was therefore a trailblazer in postgraduate cancer education. Although recently there has been a surge in postgraduate taught (PGT) courses across the UK, there are still only a few institutions focusing on cancer.

## 2 Discussion

### 2.1 Environment

UCL is consistently ranked as one of the top ten Universities globally (QS World University Ranking 2010-2025) and is currently number two in the UK for research power in the Research Excellence Framework (REF) 2021. In addition, UCL is ranked in the top 10 in the world for Life Sciences and Medicine (QS World University Ranking by subject 2025).

The importance of cancer as a discipline is recognized with the CI, where the MSc Cancer is based, being a stand-alone Division within the Faculty of Medical Sciences. The Faculty has five other Divisions, with a unique mix of pioneering research, world leading academics and clinicians, cutting edge facilities and world-renowned partner hospitals and institutions. Thus, the scope for interdisciplinary work and networking opportunities for the MSc Cancer students is extensive. Further, the CI itself is divided into departments: Cancer Biology and Pathology, Cancer Clinical Trials, Hematology and Oncology. As such our curriculum aligns with the most recent discoveries and advancements in the bench to bedside pipeline. An expanding portfolio of undergraduate and postgraduate courses, alongside the dedicated education staff within the Cancer Division has recently resulted in the creation of a new Department of Cancer Education in the CI.

### 2.2 Students

The prerequisite for the MSc Cancer course is an upper second-class Bachelor’s (BSc) degree in the life sciences or medical field from a UK university, or an overseas qualification of an equivalent standard. Our student cohort comprises of a variety of academic backgrounds, including but not limited to, degrees in Genetics, Biomedical Sciences, Molecular and Cellular Biology, Nutrition, Nursing, Dentistry and Medicine. The student enrolment on the course from the original 2009–10 cohort of 13 students, expanded by demand, to an intake of 38 students for the 2024–25 cohort (maximum cohort size 48 in the 2022–23 intake). For the last few years, the student body of home vs overseas students has remained at a 50:50 ratio and the course has attracted a good mixture of life and biomedical science graduates, medical students within the UK and clinicians from outside the UK.

### 2.3 Curriculum evolvement

**Figure 1:**
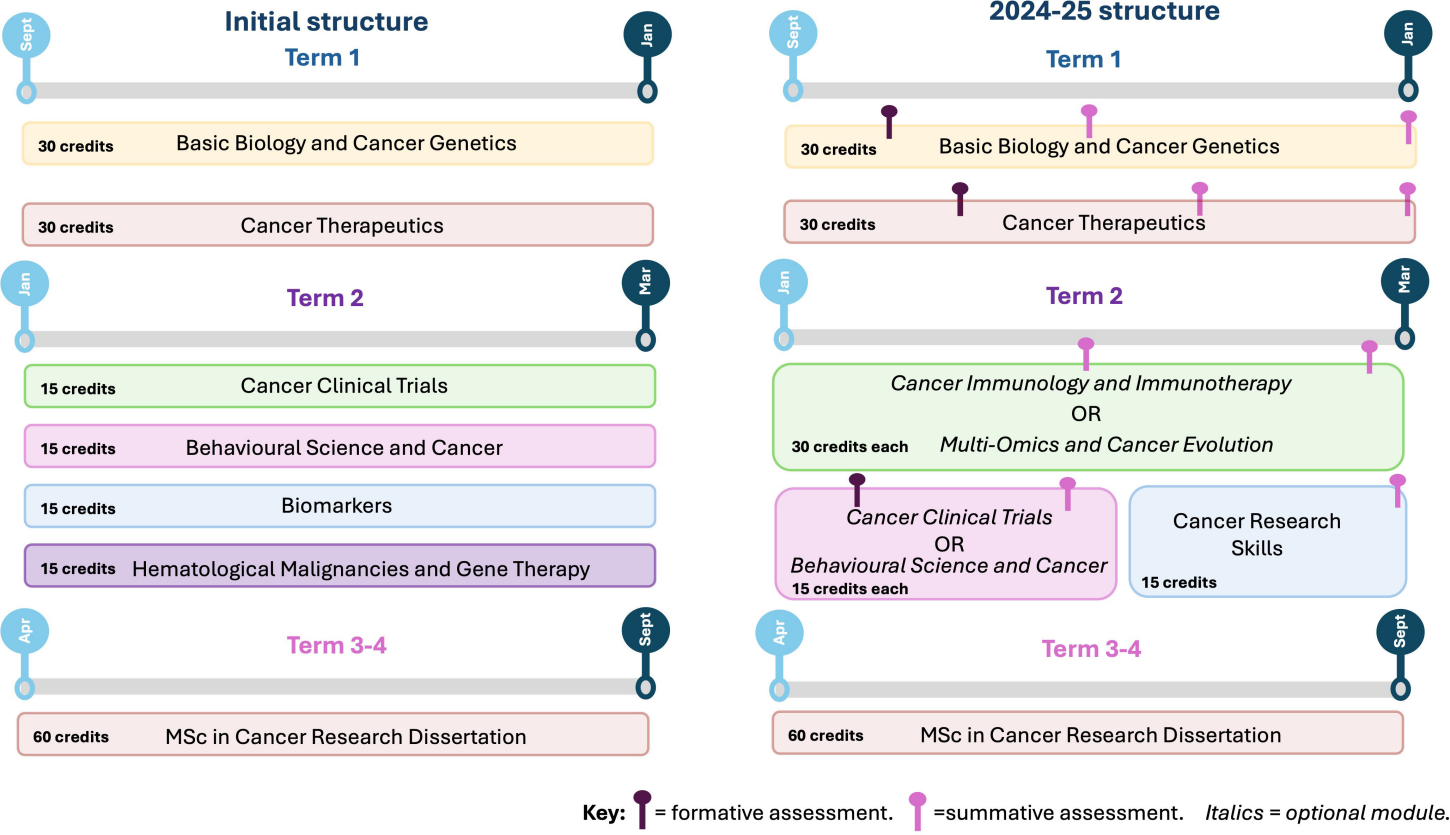
Schematic illustrating the evolvement of the 180 credit MSc Cancer course from the initial structure to the most recent. **(A)** shows the initial structure of the course with all modules being compulsory. **(B)** highlights the updated structure for the 24–25 cohort, highlighting a choice between two options modules. Choice of optional modules in term 2 are shown in *italics.* Both formative and summative deadlines are shown throughout each term for the 2024–25 intake. Figure created using Biorender.

The structure of the course has developed over time to better reflect the students’ interests and to accommodate larger course numbers (see **Figure 1**). Student input for course design was identified through student staff committees, module evaluation surveys and through informal discussions with the education team. Below describes each module for the 2024–25 curriculum in further detail.

### 2.4 Core modules: basic biology and cancer genetics

The core module ‘Basic Biology and Cancer Genetics’ was designed by the previous MSc Cancer program lead (Dr Adam Paige) around the next generation of cancer hallmarks published by Hanahan and Weinburg (3). Topics on this module cover the basics of normal molecular and cellular functions, and how these mechanisms can go awry leading to oncogenesis. Students get practical hands-on experience on how to use online databases for analysis of cancer genomes, experimental techniques used in the lab and essential skills in data analysis. Students apply their knowledge learnt in the live sessions through wet lab practical sessions and bioinformatic workshops.

The second more clinical core module ‘Cancer Therapeutics’ compliments the first, as it is devised around targeting the cancer hallmarks, in addition to advancements in precision cancer medicine. Strategically placing both these core modules in Term 1, allows all the students to recap basic biology and introduce the cohort to key concepts in cancer biology and therapeutics, ensuring all students are aligned with their understanding. These core modules are intended to give students a sufficient knowledge pool, confidence and skillset that they can take forward to the specialized and optional modules.

### 2.5 Optional modules

Firstly, in Term 2 students can choose between two 30 Credit Specialist Modules: Cancer Immunology and Immunotherapy, and Multi-Omics and Cancer Evolution. Secondly, they can choose between two 15-Credit modules: Cancer Clinical Trials, and Behavioral Science and Cancer.

Implemented due to student feedback collated from student staff committees and module evaluation surveys, the Cancer Immunology and Immunotherapy module replaced Hematological Malignancies and Gene Therapy (**Figure 1A**) as a core module. This was primarily due to the significant focus and volume of research outputs generated by the CI in this field, for example in CAR-T therapies (4). The BBC documentary ‘War in the Blood: A Cure for Cancer’ depicts the breakthroughs that UCL scientists and clinicians have made in this field in re-engineering components of the immune system for novel targeted therapies. Students learn from experts in the field on the different types of therapeutical approaches used to exploit the immune system, ranging from clinically approved standard of care to more novel approaches such as CAR-T cell therapy, antibody-drug conjugates and checkpoint inhibitors. Owning to the CI’s strong partnerships with teaching hospitals, including University College London Hospital (UCLH) and the Royal Free (RF) Hospital, students choosing this module have a unique opportunity to participate in a hospital visit. During this visit, they can see the manufacturing process of CAR-T cells for clinical applications. Having the practical experience of visualizing how bespoke immunotherapies are designed and produced at a clinical scale, significantly enhances the learning experience for the students.

Our curriculum has been updated for the 2024–25 cohort with a second optional specialist module; Multi-Omics and Cancer Evolution. This reflects the CI’s significant contributions to and international reputation in this field through high-impact research outputs, for example the ongoing TRACERx [TRAcking Cancer Evolution through therapy (Rx)] studies (5, 6). The TRACERx translational study is used as a basis for this module, allowing students to delve into the realm of dataset mining, produced from omics’ technologies and identifying key mechanisms involved in cancer evolution. What distinguishes this module is the direct involvement of field experts including senior principal investigators in computational biology and senior technicians managing the CI’s Translational Technology Platforms (TTPs), such as bioinformatics, genomics, and proteomics. This unique blend of expertise offers a bespoke teaching environment, predominantly via small group teaching and hands on workshops, immersing the students in the exciting area of precision cancer medicine.

Cancer Clinical Trials introduces the fundamental principles, design, conduct, analysis and reporting of cancer clinical trials. This module is led and taught by members of the Cancer Research UK & UCL Cancer Trials Centre (CTC) which co-ordinates both national and international clinical trials. As a result, students gain firsthand exposure to the complexities of designing, managing, and analyzing world leading clinical trials. Through this, they develop a deep understanding of trial methodologies, regulatory requirements, and the ethical considerations involved in conducting clinical cancer research. The experience also provides invaluable insights into the translational process from laboratory research to patient care.

Behavioral Science and Cancer introduces students to both behavioral and psychosocial aspects of cancer care and prevention. Students are presented with psychological theories related to health behavior change, information processing and illness representations. Students critically evaluate public awareness and understanding of cancer and its prevention, through lifestyle changes and participation in cancer screening. One strength of this optional module is that it is predominantly taught by senior researchers and clinicians outside of the Medical Sciences Faculty, which strengthens interdisciplinary links. This module expands beyond the bench to bedside pipeline and is more focused on how cancer can affect the community. Further, the module content has been updated to focus a spotlight on gaps in cancer research due to a lack of representation of marginalized communities, and how we can collectively address these issues to increase representation across the community, which is a top priority for cancer research (7, 8).

### 2.6 Core modules: cancer research skills and research dissertation

The final core 15 Credit taught module is Cancer Research Skills. Unlike the traditional content-heavy module, this module’s primary aim is the development of transferable skills essential for employability. Learning on the module is focused on critically evaluating published papers and grant applications, defining research hypotheses and aims, evaluating data quality and application of fundamental statistical methods in data analysis. Further, we offer ample opportunity for students to understand the basic principles of scientific communication. This module closes Term 2, students are holistically and academically prepared to embark on their Research Dissertation.

The MSc Cancer course concludes with the Research Dissertation that runs through Term 3-4. This 60 Credit module allows students to consolidate their theoretical knowledge and practical skills and apply these to a specific research question. Students employ the scientific method needed to address this question, through data collection and analysis, either in a wet laboratory, bioinformatic or clinical research group. This research modules requires collaborative working within a research team, enabling students to implement professional and employability skills needed for a successful research career. Our course also offers cancer research projects outside of the CI, for example, in collaboration with the Faculty of Engineering, the Sarah Cannon Institute or with clinicians in partnering hospitals, highlighting the interdisciplinary nature of our course.

**Figure 2:**
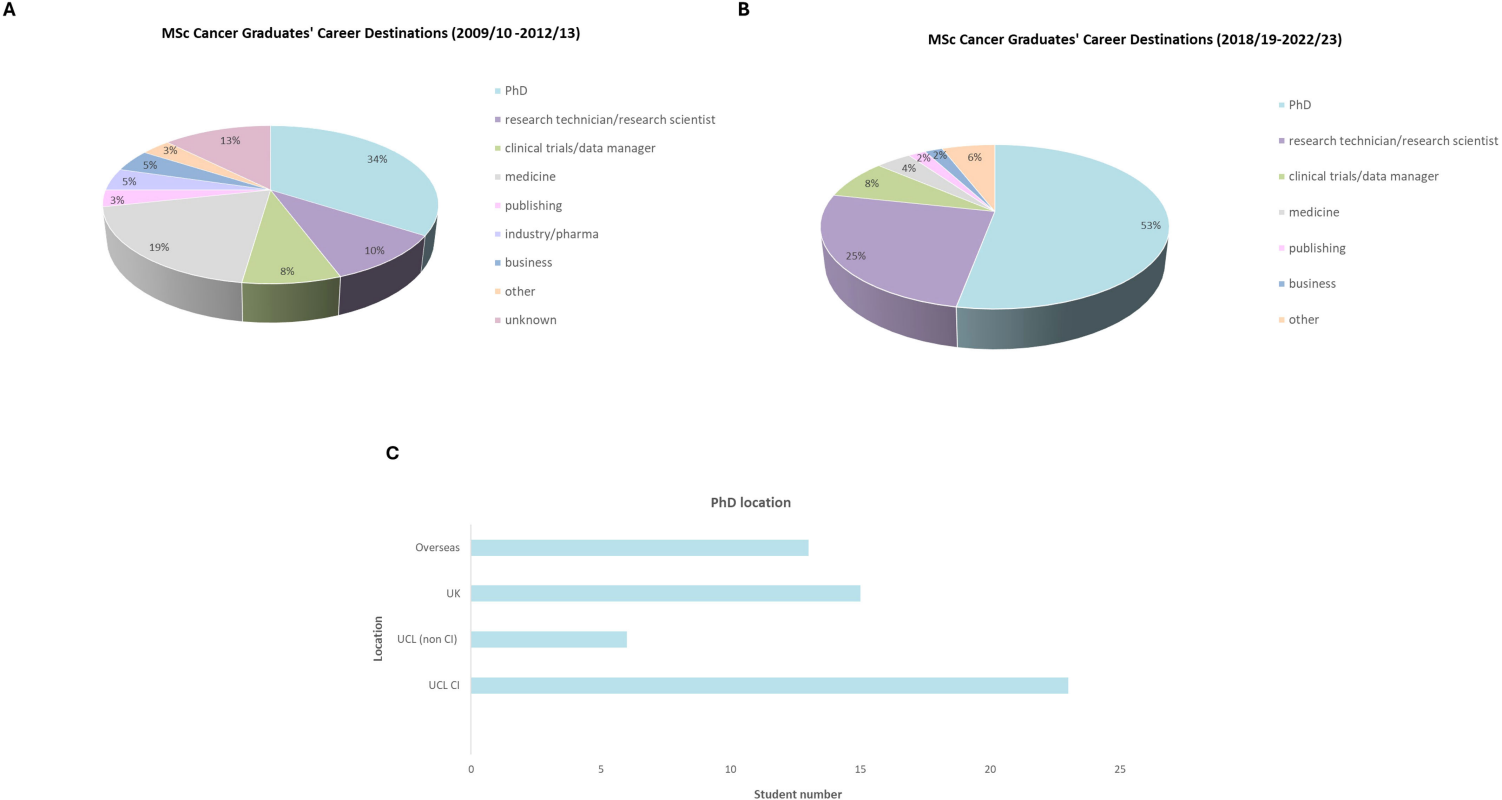
MSc Cancer student graduate career data, collected from 2009–13 and 2018-23. **(A)** data collected for the first four cohorts showing the sector of employment or further education. 88 students responded. **(B)** data collected for the most recent five cohorts showing the sector of employment or further education. 51 students responded. **(C)** PhD geographical location (total 57 students).

Over the past 15 years, the quality of research dissertations produced by MSc Cancer students has been outstanding, with significant contributions in areas such as immunotherapies and cancer evolution, proteomics and cancer cell signaling, evidenced by high quality publications such as (9, 10), and (Tibarewal et al., 2024 – manuscript in preparation). The program offers numerous research and networking opportunities, with some students progressing to PhD projects, providing preliminary data for grant applications or securing positions as research assistants/technicians within the same research group (see **Figure 2**). Notably, one student was inspired by a summative assessment from the taught modules, which led to a publication on cancer vaccines in *Immunotherapy Advances* (11). Ultimately, the CI is a world leading research hub where groundbreaking discoveries are changing the landscape for more effective and precise cancer therapies, and the work produced by the MSc Cancer students contributes to this research.

### 2.7 Educational practices

The higher education sector is becoming increasingly competitive, where students are more considered when choosing postgraduate degrees (12). The quality of teaching and learning on a course can heavily influence students’ informed decisions and applying for a course. Thus, it is imperative to maintain an exceptional level of teaching practices, to retain student numbers and continue to grow the MSc Cancer community.

The educational practices carried out on the MSc Cancer course, are a dynamic blend of both theoretical learning and practical applications. This fosters an environment where students can strengthen their critical thinking skills and professional competence. We are acutely aware of the diversity across learner types in our student body, thus we embed varied teaching practices across our program to ensure all learners’ needs are met. Teaching practices include interactive lectures, hands-on workshops and lab practicals, small group teaching, research seminars and asynchronous learning. Assessments across the program are meticulously planned to ensure the assessment load is manageable for the students and there is minimal overlap with deadlines (as shown in **Figure 1B**). Further, the innovative summative assessments comprise of different strategies to assess student learning, for example data analysis coursework, examinations, pitching of a novel biomarker as an oral presentation and critical evaluation of clinical trials.

Universities have a responsibility to provide opportunities to equip students with a skillset that goes beyond subject specific knowledge that are highly sought after from employers (13). Thus, constant innovations in assessment modalities are needed to meet these demands. An additional type of formative assessment was recently introduced into the curriculum (2023–24 cohort), where students had the opportunity to peer review one another’s work. Opportunities for students in higher education to participate in peer review is scarce, however the collaborative learning process of peer review offers students diverse learning benefits (14). For example, peer review encourages students to take a reflective role in learning, which has shown to help promote higher cognitive skills and harness in on their problem solving skills by identifying strengths and weaknesses of their peer’s work (15). This aids our student cohort transitioning from passive learners to active, while also enhancing their employability skills (16). The evidence that our educational practices are working is reflected by the results from the internal Annual Program Survey, which was launched by UCL in 2023. Data collated from the 2022–23 and 2023–24 student cohorts highlight that the course scored 86.5% on ‘teaching and learning’ and 96.2% on ‘my program is up to date and informed with current thinking’ (29% response rate).

### 2.8 Career prospects

The MSc Cancer offers excellent career prospects for graduates and has provided a foundation for securing job roles in academia, industry and medicine. The course has also facilitated pathways to PhD opportunities, both nationally and internationally. This is evidenced by our in-house graduate questionnaire (**Figure 2**), which collected granular insights from the first four and most recent five cohorts of MSc Cancer graduates to understand the course’s impact on their careers.

The data in **Figure 2** indicates that the MSc Cancer course continues to provide fruitful opportunities for further education and employment. There is a particular strong correlation between MSc Cancer graduates and securing PhD positions, particularly within UCL.

Data from the most recent five cohorts showed that 88.7% of graduates either agreed or strongly agreed that completing the MSc Cancer helped secure their current job role or PhD position (data available upon request). This emphasizes that the curriculum, educational practices and research opportunities provide a secure foundation for an array of career prospects.

The below quote from our 2022–23 cohort perfectly captures what we have tried to achieve with the course:

*‘While it was academically challenging, the course allowed me to learn more than just academic and research skills. Throughout the program, I learned how to be able to connect scientific knowledge with real-life applications through scientific communication, how to build my networking skills, and how to improve myself to become a more effective student. While in MSc Cancer, I found it fascinating that there’s a plethora of compelling niche fields related to cancer research. A career is a journey to discover, and I believe my experience in MSc Cancer will be a strong foundation for my future endeavors’.*

## 3 Conclusion

Overall, despite economic challenges and the changing landscape of postgraduate education, the MSc Cancer has continued to thrive during its 15 successful years. The curriculum embeds key academic and transferable skills that fully equip our graduates to progress onto a career within academia or outside, evidenced by our graduate careers data. We will continue to produce high quality research outputs and implement advancements in our curriculum and teaching practices, ensuring the MSc Cancer Course at UCL remains a pioneering postgraduate degree.

## Data Availability

The original contributions presented in the study are included in the article/supplementary material. Further inquiries can be directed to the corresponding author.
